# Comparative Transcriptome Reveals Benzenoid Biosynthesis Regulation as Inducer of Floral Scent in the Woody Plant *Prunus mume*

**DOI:** 10.3389/fpls.2017.00319

**Published:** 2017-03-10

**Authors:** Kai Zhao, Weiru Yang, Yuzhen Zhou, Jie Zhang, Yushu Li, Sagheer Ahmad, Qixiang Zhang

**Affiliations:** Beijing Key Laboratory of Ornamental Plants Germplasm Innovation and Molecular Breeding, National Engineering Research Center for Floriculture, Beijing Laboratory of Urban and Rural Ecological Environment, Key Laboratory of Genetics and Breeding in Forest Trees and Ornamental Plants of Ministry of Education, School of Landscape Architecture, Beijing Forestry UniversityBeijing, China

**Keywords:** Mei, floral scent, transcriptome, benzenoid biosynthesis, transcription factors

## Abstract

Mei (*Prunus mume*) is a peculiar woody ornamental plant famous for its inviting fragrance in winter. However, in this valuable plant, the mechanism behind floral volatile development remains poorly defined. Therefore, to explore the floral scent formation, a comparative transcriptome was conducted in order to identify the global transcripts specifying flower buds and blooming flowers of *P. mume*. Differentially expressed genes were identified between the two different stages showing great discrepancy in floral volatile production. Moreover, according to the expression specificity among the organs (stem, root, fruit, leaf), we summarized one gene cluster regulating the benzenoid floral scent. Significant gene changes were observed in accordance with the formation of benzenoid, thus pointing the pivotal roles of genes as well as cytochrome-P450s and short chain dehydrogenases in the benzenoid biosynthetic process. Further, transcription factors like EMISSION OF BENZENOID I and ODORANT I performed the same expression pattern suggesting key roles in the management of the downstream genes. Taken together, these data provide potential novel anchors for the benzenoid pathway, and the insight for the floral scent induction and regulation mechanism in woody plants.

## Introduction

Presence of volatiles in the flowers has been a special attraction for humans since antiquity and with time this appeal has permeated several aspects of man's life. As a matter of fact, they have become integral constituents of cosmetics, perfumes, medicinal products, and flavorings. However, they are primarily involved in maintaining the ecological bridging of flowers to that of diverse range of visitors (florivores, pollinators, pathogens), thereby playing a significant role in reproductive success and evolutionary variability in plants. The intricate biochemical pathways behind their synthesis are driven by both internal and external factors, thus enabling the controlled spatial and temporal emission of floral volatiles (Muhlemann et al., 2014). Persuasive floral scent by these volatiles is the most demanding trait in ornamental plants as an esthetic preference for consumers and an attraction for pollinators as well (Pichersky and Dudareva, 2007; Raguso, 2008). Complex lipophilic molecules with low-molecular weight determine the quality of floral scent (Dudareva and Pichersky, 2008) and to date, more than 1,700 floral scent related compounds have been recognized which are synthesized through terpenoid, phenylpropanoid/benzenoid, and fatty acid biosynthetic pathways (Knudsen et al., 2006; Muhlemann et al., 2014).

Mei (*Prunus mume Sieb. et Zucc*.) is an important fruit tree in China and other East Asian countries for its economic value (Chen, 1996). It has been domesticated with favorite ornamental characteristics, incorporating colorful corollas, varying types of branches, and particularly inviting fragrance (Sun et al., 2013; Xu et al., 2014; Zhang et al., 2015). However, little is known about the molecular mechanisms that control flower aroma formation in this fantastic species. Mei flowers produce a strong aroma of benzenoids, and benzyl acetate protrudes from the dominant compounds. This endows *P. mume* with an excellent advantage from the most interspecific hybrids and other species of *Prunus*.

Floral scent emission is often restricted to particular flower tissues which is believed to be regulated at the level of scent biosynthetic gene expression and enzyme activity. The biosynthesis of phenylpropanoid/benzenoid compounds has been described synoptically with the discovery of core enzymes and the exhaustive β-oxidative pathway in petunia (Qualley et al., 2012). In different species, the floral scent of benzenoids tends to be various owing to the modification in the final step such as methylation, hydroxylation, and acetylation (Boatright et al., 2004; Klempien et al., 2012). Benzyl acetate was reported to be synthesized from benzyl alcohol in *Clarkia* (Nam et al., 1999). However, the formation of part precursors like benzaldehyde remains uncharted.

Presently, RNA sequencing is an important application to analyze the mechanism controlling unique plant feature formation at transcriptional levels (Bar-Akiva et al., 2010; Onda et al., 2015). It helps researchers find novel gene functions and set their interaction among huge data. Though the lack of reference genome is one of the limitations for many scented plants, such as ylang ylang, *syringa oblate*, and winter sweet, RNA-seq profiling still provides an instrument to measure large scale transcript changes in normalized samples and allows the inter comparison to recognize core regulatory genes (Liu et al., 2014; Jin et al., 2015; Zheng et al., 2015). Especially for the benzenoid type scented plants, the differentially expressing genes depict an outline for the biosynthesis of benzenoids in each species. Meanwhile, several transcriptional factors (TF) are also mentioned in the regulation of benzenoid biosynthesis of petunia flowers. ODORANT1 is the first identified TF to guide the flux in shikimate pathway, and EMISSION OF BENZENOIDS I and II positively regulate the phenylpropanoid/ benzenoid pathway (Verdonk et al., 2005; Spitzer-Rimon et al., 2010). Recently, the interplay of these three MYB TFs has been demonstrated (Van Moerkercke et al., 2011; Fenske et al., 2015).

Since the complete genome sequencing in 2012, scent formation has been regarded as an important character to study in *P. mume* (Zhang et al., 2012). RNA-seq studies demonstrate vigorous strength to identify the putative genes specifying the biosynthesis of benzenoid precursors and regulatory mechanism in this volatile species. In this study, a comparative transcriptome of Mei flower was constructed by two transcription datasets for developed flower buds and blooming flowers. Differentially expressed genes (DEGs) were first extracted and narrowed by associating transcriptomes of different organs (from leaf, fruit, stem, and root). Specially expressed genes encoding transcription factors and enzymes like short-chain dehydrogenases/reductases (*SDRs*) and cytochrome P450s were highlighted. These datasets could be applied as significant resources to supplement benzenoid metabolic processes and regulation mechanism. Furthermore, they would furnish vital clues to uncover other biological mechanisms in nearby species and scent-related plants.

## Materials and methods

### Plant materials collection and RNA extraction

**Figure 1:**
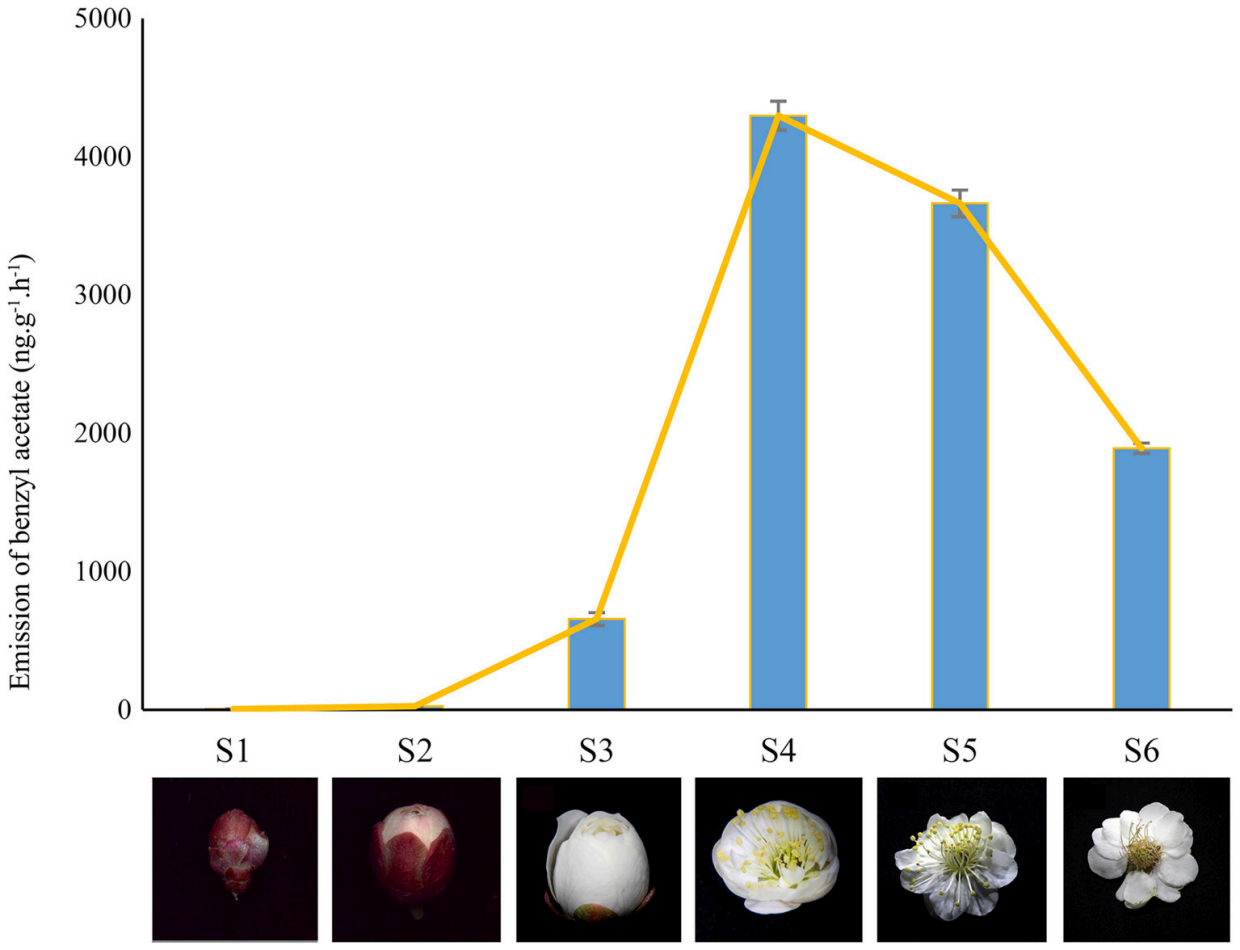
**Different developing stages from *P. mume* (S1-S3) Developed bud; (S4) Squaring flower; (S5) fully blooming stage; (S6) fading stage**. The flowers in stages of S2 and S4 were used to pool cDNA libraries for Illumina sequencing.

Plant materials were obtained from *P. mume* “Sanlun Yudie” at the Beijing Forestry University, China (116.3°E, 40.0°N). The samples were collected at fixed daytime (10:00 am) to differentiate the aroma changing phase including flower buds at different expanding stages (well-developed, S1–S3) and blooming flowers (S4–S6) with typical Mei scent as shown in Figure 1 (Chen, 1996; Wang et al., 2014). Immediately after collection, samples were frozen in liquid nitrogen, and stored at −80°C. Total RNAs of two stages (S2 and S4) were extracted applying RNAisomate RNA Easy spin Isolation System, and were assessed using NanoDrop ND2000 (Liu et al., 2015). A260/A280 was 1.8~2.2,28S/18S>1.0, RNA concentration≥400 ng/μL.

### Illumina sequencing and reads mapping

To reveal the difference between non-scent buds and scented blooming flowers, equivalent quantities of RNA were isolated from same stages from three trees, each individual contained 5–10 flowers. Then sample from same stages were mixed to average the difference between individuals. Total of 10 μg RNA samples were selected to construct comparative sequencing pools of S2 and S4 stage, respectively. Preparation of the cDNA library was applied in detail to construct two libraries, Bud and Flower, for comparative transcriptome sequencing. Then, sequencing was performed by Illumina HiSeq™2000 platform (BGI, Shenzhen, China) and 100 bp paired-end format raw reads were generated according to the manufacturer‘s instructions by base calling (Illumina Inc. San Diego, CA). After filtering raw reads (adaptor sequences, empty reads, reads with unknown nucleotides larger than 5% and ambiguous N), we employed clean reads for mapping the *P. mume* genome v.1.0 (http://prunusmumegenome.bjfu.edu.cn/). Mapping of the clean reads was carried out with SOAP2 under default parameters allowing 5 mismatches at the most (Hurgobin, 2016). The reference gene was defined as an expression gene, if at least one clean read showed unique match. P-fam database was attained from the reference annotation of the *P. mume* genome. This comparative transcriptome was deposited in the Genome sequence archive (GSA). The accession numbers were PRJCA000274. And the associated datasets involving different organs (leaf, root, stem, fruit) were acquired from NCBISequence Read Archive (SRA) with accession numbers of SRP014885.

### DEG selection, expression patterns analysis, and function annotion

Differentially expressed genes were primarily detected by the software of edgeR. Then, the fold change [absolute value of log_2_ (fold change)> 1] and FDR ≤ 0.05 was used as the threshold to determine significant differences in gene expression (Audic and Claverie, 1997; Mariani et al., 2003). Transcripts per million (TPM) was used to provide normalized values of gene expression that enabled transcript comparisons between samples While the variations in gene length and the size of library matters (Li and Dewey, 2011).

For the DGEs, latest Gene Ontology (GO) annotations were obtained using Blast2GO v.2.3.5 with the default parameters. GO terms of DEGs were significantly enriched by gene set enrichment analysis (GSEA) with the correct *P* < 0.05.

HMMER 3.0 software with a threshold *E*-value of 10^−5^ was applied to search against the databases (Finn et al., 2015). Transcription factors were labeled under iTAK (http://bioinfo.bti.cornell.edu/cgi-bin/itak/index.cgi) based on conserved domains of genes (Zheng et al., 2016). For the pathway exhibition, MapMan analysis were performed by calculating the fold change of the genes (Klie and Nikoloski, 2012; Ramsak et al., 2014; Chandran et al., 2016). Sequences of DGEs were uploaded and annotated by Mercator (http://www.plabipd.de/portal/mercator-sequence-annotation). Final display and automatic application of the Wilcoxon rank sum test were conducted by MapMan version 3.6.0.

Subsequently, the differentially expressed TFs were picked out from DEGs. STEM (Short Time series Expression Miner) software (http://www.cs.cmu.edu/~jernst/stem/) was used to gather the specially expressed DEGs and distinguished the temporal expression profiles (Ernst and Bar-Joseph, 2006).

### qRT-PCR verification

Experimental method for quantitative reverse transcription-PCR was applied as formerly described (Zhou et al., 2016). The primers for qRT-PCR were designed with Oligo8 software (Supplementary Table 1). Total RNA was extracted from samples using Trizol reagent (Invitrogen, USA) following the manufacturer's instructions. RNA was treated with RNase-free DNase (Promega, USA) to remove residual genomic DNA. Firststrand cDNA was synthesized from 2 μg total RNA using a TIANScript First Strand cDNA Synthesis Kit (Tiangen, China) according to manufacturer's instructions. Realtime RT-PCR was conducted using the PikoReal real-time PCR system (Thermo Fisher Scientific, Germany). Reactions were carried out in a 20 μl volume containing 0.5 μl cDNA, 400 nM each primer, and 10 μl SYBR Premix Ex Taq II (Takara, China) with the following conditions: 30 s at 95°C, 40 cycles of 5 s at 95°C, and 30 s at 60°C. Melting curve analysis was conducted to test the specificity of each primer pair. All qRT-PCR experiments were implemented with three biological replicates, and each replicate was analyzed in triplicate. The relative expression levels were calculated using the 2^−ΔΔCt^ method, with the protein phosphatase 2A (PP2A) gene of *P. mume* as the reference gene (Wang et al., 2014).

## Results

### Datasets evaluation

In the aggregate, two sample pools yielded 24.9 and 24.8 million reads corresponding to over 2.2 billion basepairs each sample (Table 1). Valid quality scores of the reads were received with balanced nucleotide distribution. We achieved 76.81 and 61.43% genome map ratios in bud and flower, respectively. The detailed quality scores across all bases and the mapping information for both pools were separately listed (Supplementary Figure 1). A total of 22,429 expressed genes were gathered in both libraries and 19,511 expressed genes emerged in both pools. As a whole, TPM got max values of 139,727 and 352,186 in both stages, respectively. There were 90 genes with TPMs above 5,000 in the bud, while 237 in the flowers.

**Table 1:** **Statistics of the reads obtained and their mapping information on the genomes of Mei flower**.

**Library name**	**Total reads**	**Genome map rate (%)**	**Gene map rate (%)**	**Expressed Gene**	**Perfect match (%)**	**Unique match (%)**	**Unmapped reads (%)**	**Max TPM**
Bud	24,961,904	76.81	64.48	21,050	48.28	73.65	23.19	139,727
Flower	24,885,518	61.43	63.10	20,890	37.66	58.92	38.57	352,186

Except the heads and tails, reads were distributed uniformly on the gene body, indicating better sequencing randomness which availed the next analysis. To estimate gene expression, eight housekeeping genes covering *Actin* and *PP2A* were selected (Wang et al., 2014). Based on pairwise comparisons of the two stages, none of these reference genes could give obvious differential expression, with log_2_ (fold change) ranging from 0.14 for *Translation enlongation factor 2* to 1.73 for *Ubiquitin*. As for the common reference genes of *P. mume*, the TPM of Proteinphosphatase2A-1 stayed around 432–546. The expressions of housekeeping genes remaining stable between different pools illustrated accuracy of this transcriptome, and met the requirements for further analysis (Table 2).

**Table 2:** **Expression of eight selected housekeeping genes**.

**Gene name**	**Conserved domain**	**Locus tag**	**Query**	**Length**	**Coverage**		**TPM**		**log_2_FD**
					**Bud**	**Flower**	**Bud**	**Flower**	
Actin2/7	actin	Pm005252	AT5G09810	906	98.90	98.90	155	193	0.31
PP2A-1	ProteinPhosphatase 2A	Pm029033	AT1G13320	1764	99.72	99.89	432	546	0.33
PP2A-2	Protein phosphatase 2A	Pm006362	AT1G59830	921	99.78	99.78	627	1359	1.11
SAND	SAND family protein	Pm001035	AT2G28390	1833	99.18	98.87	126	225	0.83
TEF2	Translation enlongation factor 2	Pm011035	AT1G56070	2532	99.92	99.92	2690	2983	0.14
TUA	Alpha Tubulin-5	Pm000088	AT5G19780	1353	99.19	99.04	1760	2797	0.66
UBQ	Ubiquitin	Pm009747	AT4G02890	1599	100.06	100.06	2300	7639	1.73
UBC	Ubiquitin conjugating enzyme E2	Pm024097	AT5G53300	447	98.66	99.33	3849	8452	1.13

### Screening of DEGS associated with scent emission

**Figure 2:**
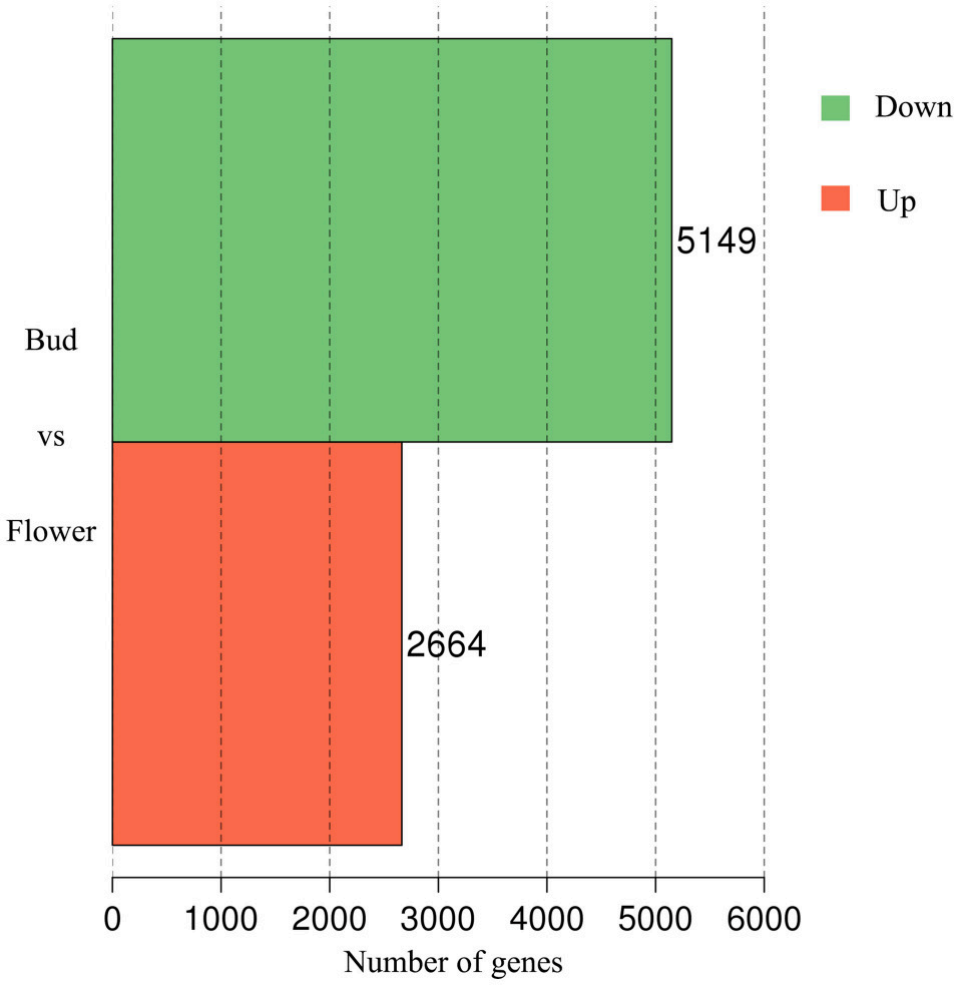
**The DEGs of comparative transcriptome**. The red block displayed 2,664 up-regulated and the green one established 5,149 down-regulated genes.

In accompany with the flower bud development, benzyl acetate in Mei flower presented vast accumulation, from S2 to S4, then decreased at S5–S6. To identify the DEGs in both developmental stages, we calculated the number of clean tags for each gene, and the DEGs between the two stages were detected according to the described method. In the library of Flower compared to Bud, a total of 7,813 DEGs were exposed, containing 2,664 up-regulated and 5,149 down-regulated genes (Figure 2). Most of these genes declined in the blooming flower suggesting that there were more complex activities in buds (Supplementary Figure 2, Supplementary Tables 3,4). Furthermore, 401 and 356 genes expressed uniquely in flower and bud, respectively, and 7,056 genes, about 90.31% of all the DEGs, appeared in both libraries, but at different levels.

**Figure 3:**
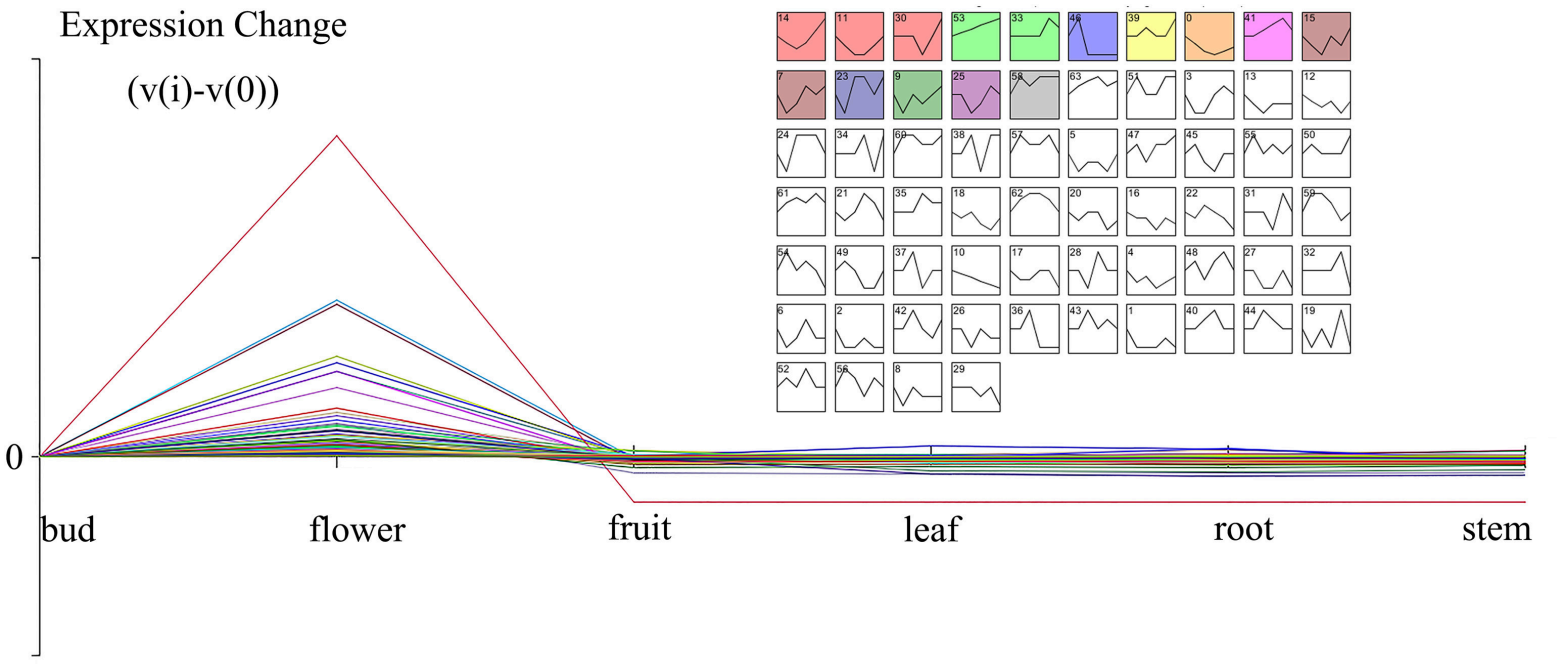
**The enlarged cluster 46 (Blue block, *p*-value 6.5E-11) showed the scent related genes with a pattern of flower organ specificity**. Clusters ordered based on number of genes and profiles ordered by significance.

The gene expressions of five organs (bud, fruit, leaf, root, and stem) were manifested from the former transcriptome datasets under the same platform. In combination with the results of different organs transcriptome, all DEGs were classified by STEM cluster analysis. The cluster analysis of DEGs disclosed 64 expression profiles, with 11 remarkable profiles (Figure 3). The genes specially expressed in the bud and flower displayed potential correlation with the emission of volatile compounds. Cluster 46, *P*-value 6.5E-11, gathered this pattern profile (0,1,-1,-1,-1,-1) with 681 genes representing that these genes started expressing from buds, gained peak expression in the flower and showed low expression in other organs.

### Gene annotation and classifications for the DEGS

**Figure 4:**
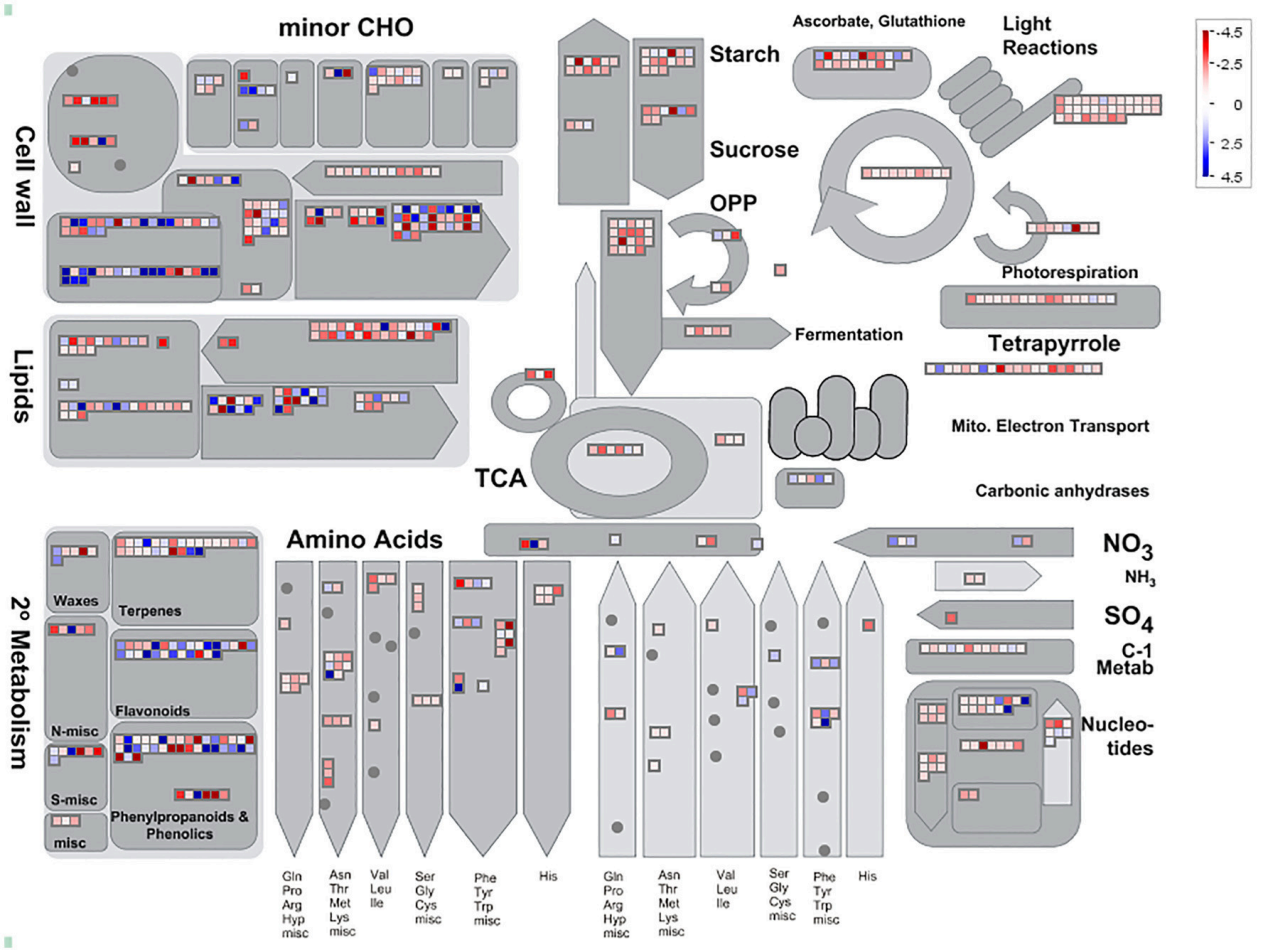
**Analysis of candidate genes using MapMan**. Metabolism Overview revealed candidate genes assigned to 16 processes: cell wall metabolism, minor carbohydrate metabolism, starch metabolism, sugar metabolism, photosynthesis including light reactions, tetrapyrrole, Calvin Cycle, and photorespiration, glycolysis, metabolism, TCA Cycle, oxidative pentose phosphate pathway, mitochondrial electron transport, amino acid biosynthesis, nucleotide metabolism, lipid metabolism, and secondary metabolism. The blue diamonds mean up-regulated, otherwise in the red.

**Figure 5:**
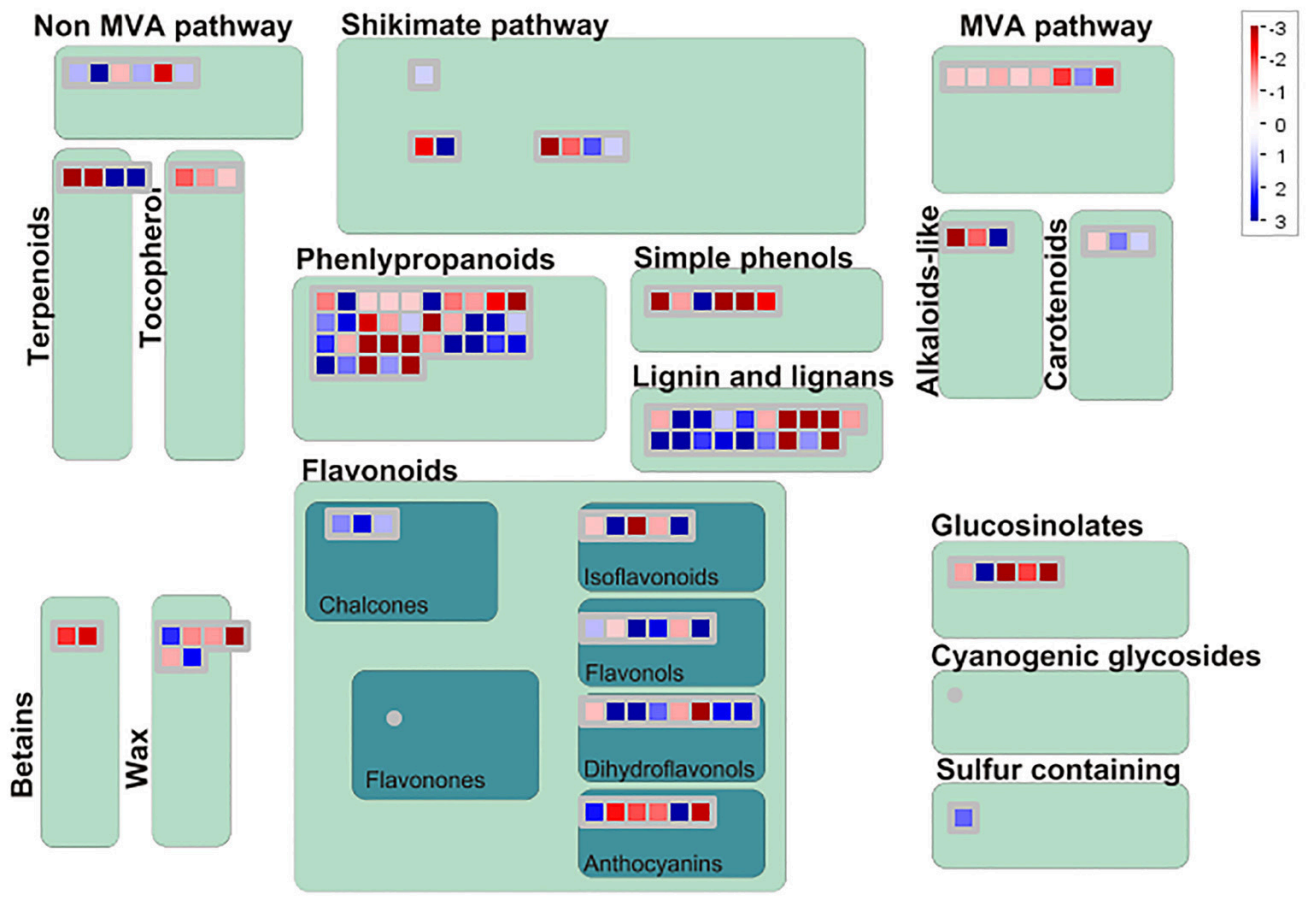
**Display of secondary metabolism by Mapman**. DEGs focus on the pathway of phenlypropanoids, Lignin and lignans, shikimate etc. The blue diamonds mean up-regulated, otherwise in the red.

Among 7,813 DEGs, approximately 5,089 genes could be annotated in GO database by sequence homologies, while 6,878 genes got hit in *Arabidopsis thaliana* (Supplementary Figure 3) with an overview fabricated by MapMan (Figure 4). As shown in the metabolism overview map, most DEGs lay on the subareas of second metabolism, lipids metabolism, and cell wall metabolism. Between the two stages from bud to blooming, the cells of Mei flower seemed to accomplish the division and 159 genes out of 417 still function in the development pathway of cellular response. The changes of cell function concentrated on the protein degradation and posttranslational modification. And proteins in many large enzyme families broke like Cytochrome P450, UDP glycosyltransferases, and peroxidases. The secondary metabolic processes were expounded in detail (Figure 5), and enriched in pathways like phenlypropanoids, lignin, and lignans, dramatically.

**Figure 6:**
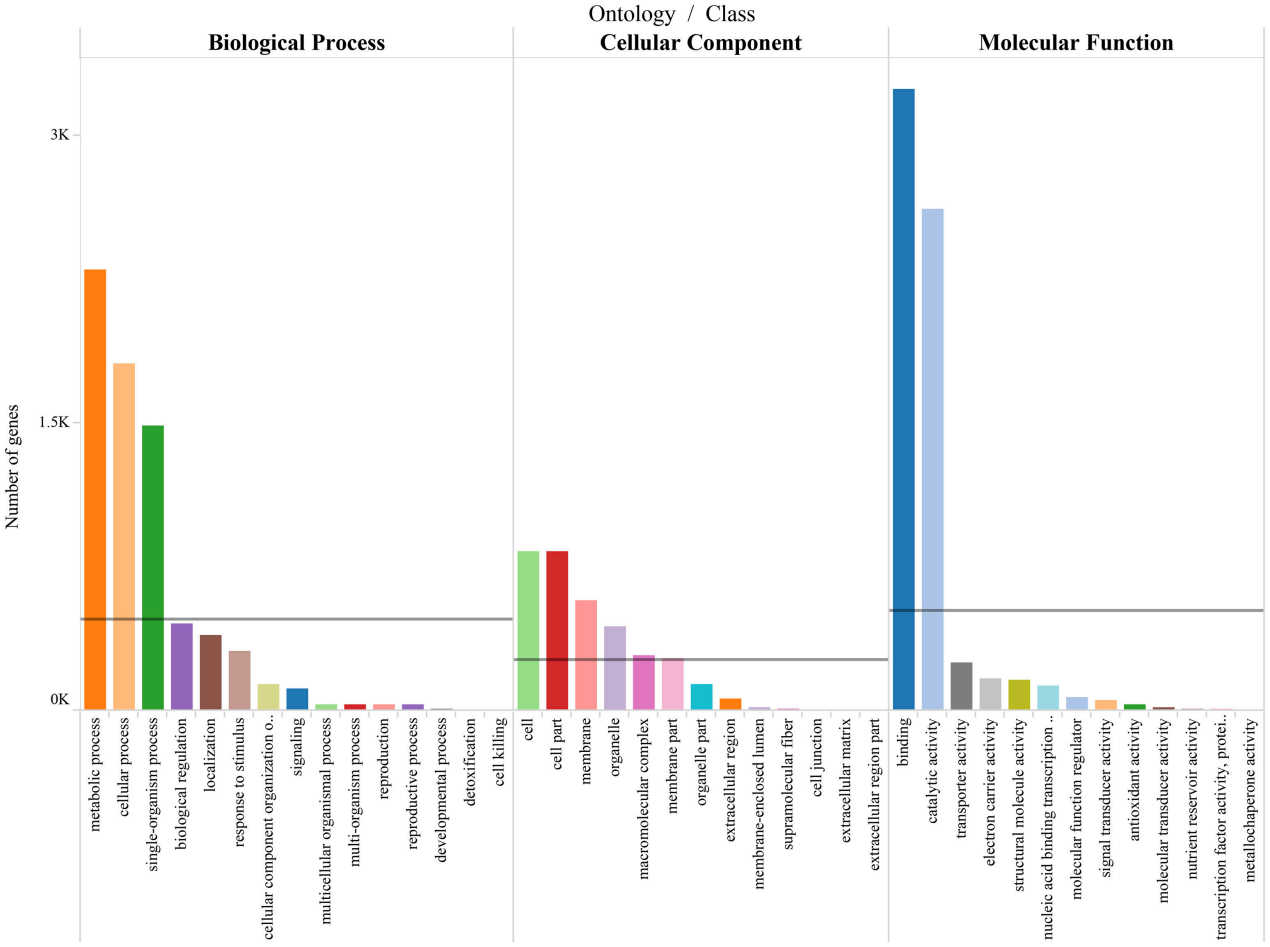
**Gene ontology categories of *P.mume* DEGs**. The results are summarized in mainly three categories: biological process, cellular component and molecular function. The lines reveal the averages of genes in each category.

To differentiate the functions of the separated genes, GO enrichment analysis was performed (Supplementary Table 5), and the predominant GO categories are presented (Figure 6). In the categories, 5,089 annotated DEGs were divided into 41 functional GO subcategories within the three main ontologies (biological processes, cellular component, and molecular function). In the biological process ontology, metabolic processes and cellular processes possessed the top two represented GO categories, with 2,301 and 1,814 genes, respectively. In addition, 1,485 genes were annotated to single-organism process. GO categories “Cell” and “Cell part” ranked the top two in the cellular component ontology, with both 829 genes. And “membrance” and “organelle” possessed 577 and 441 respectively. These genes were associated with cell cycle control, cell division, and chromosome partitioning. In the molecular function ontology, a mass of genes were annotated to the categories of Binding and Catalytic activity (3,248 and 2,622). Two hundred forty-six genes were annotated to transporter activity. The GO annotations of these genes might aid in the identification of the molecular mechanisms of floral scent production and emission.

### Analysis of proposed genes related to known benzenoid biosynthesis

To highlight the pathway of benzenoid biosynthesis in *P. mume*, and recognize the functions of committed genes in this pathway, the pivotal encoding enzymes were sought by the phmmer search from HMMER 3.0.

**Figure 7:**
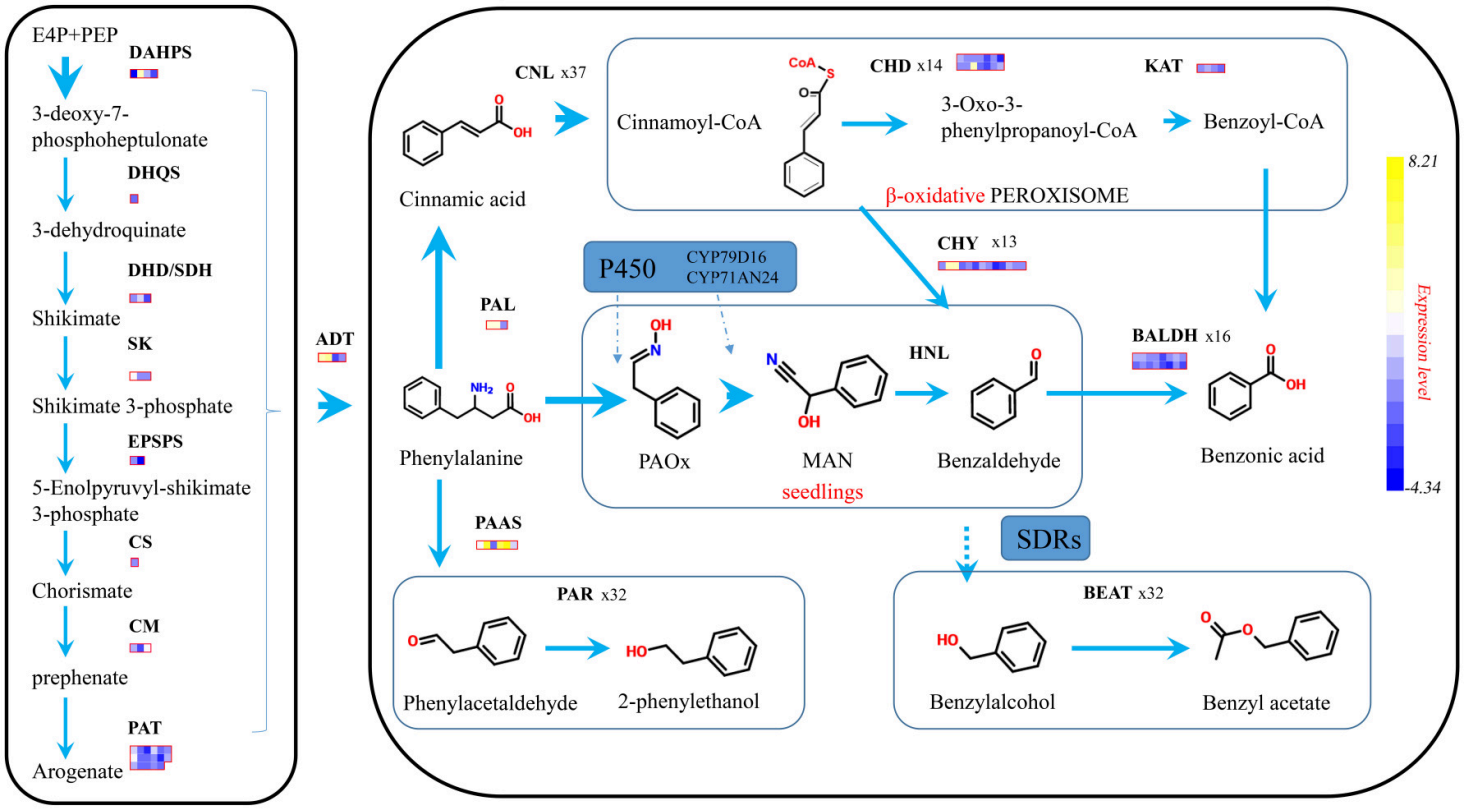
**Proposed schematic representation of phenylpropanoid/ benzenoid biosynthetic pathway**. Phenylpropanoid/ benzenoids are derived from phenylalanine, which itself is synthesized via the shikimate biosynthetic pathways. Larger arrows indicate there exist genes with strong expression level above 5 thousand of TPM. Each block means a fold change for a single gene.

The biosynthesis of benzenoids in plant usually starts from shikimate and through arogenate pathways to form phelalanine, which crosses the boundary between primary and second metabolism (Figure 7). The seven reactions catalyzed by six enzymes formed the shikimate pathway, and transforms phosphoenolpyruvate (PEP) with erythrose 4-phosphate (E4P) to chorismate (Tzin and Galili, 2010). Fourteen shikimate pathway genes were spotted in the *P. mume* genome, possessing four 3-deoxy-7-phosphoheptulonate synthase (*PmDAHPS*), one 3-dehydroquinate synthase (*PmDHQS)*, three dehydroquinate dehydratase/shikimate dehydrogenase (*PmDHD/SHD*), three shikimate kinase (*PmSK*), two 3-phospho shikimate 1-carboxyvinyltransferase genes (*PmEPSPS*), and one chorismate synthase gene (*PmCS*). For these genes, two out of four *PmDAHPS*s displayed enormous expression difference. The TPM of *PmDAHPS1* declined from 5,646 to 788, and that of *PmDAHPS2* rose dramatically from 1,508 to 50,480. As for other genes along the pathway, the expression kept low or declined. The TPM levels of these genes at both stages were declared in detail (Supplementary Table 2).

After the formation of chorismate, phenylalanine was synthesized under the catalysis of three chorismate mutases (*PmCMs*), 17 prephenate aminotransferase (*PmPAT*) genes, and four arogenate dehydratases (*PmADTs*) in *P. mume*. In four *ADT* genes, only *PmADT1*and *PmADT2* were ascending significantly at the flower squaring process (Figure 7). And the peak TPM of *PmADT2*, around 13,686, was almost 2-fold more than that of *PmADT1*.

*PAL*, which is recognized as beginner/precursor of benzenoid biosynthesis, deaminates phenylalanine to cinnamic acid (Zhang and Liu, 2015). Three *PmPALs* existed in the *P. mume* genome and two of them were significantly regulated with TPMs 35,788 and 3,648 in the blooming flower. Then, phelalanine (C3 type) was split toward different pathway to synthesize phenylpropanoid-related compounds (C2 type) or benzenoids (C1 type). In the flower of rose, phenylalanine was converted into phenethyl alcohol by the way of phenylacetaldehyde, under two enzymes, *PAAS* and *PAR*. In *P. mume*, 6 *PmPAAS*s were found, and only one got the max TPM 4,553 and 3.51-fold change in squaring stage. When *PAR* was used as query to find the homologous genes in *P. mume*, 32 candidate *PmPAR*s heaved out and two of them stated high transcription in blooming stage.

The CoA-dependent β-oxidative pathway, which catalyzes trans-cinnamic acidinto benzoic acid (BA) in petunia flowers, implicates three enzymes. We used the characterized petunia genes as baits to search of *P. mume* genome, 37 Cinnamate:CoA ligase/acylactivating enzyme (*PmCNL/AAE*), 14 cinnamoyl-CoA hydratase dehydrogenase (*PmCHD*), and four 3-ketoacyl CoA thiolase (*PmKAT*) were found. The expression pattern of two *PmCNL* genes showed highly positive correlation with the emission of volatile benzenoids. *PmCNL1* possessed an almost 6-fold change from 113 to 10,117, and *PmCNL6* kept high TPM from 5,069 to 12,970. For *PmCHDs* and *PmKATs*, the expression from bud to blooming stage showed insignificant change.

Benzaldehyde dehydrogenase (*BALD*) was reported to account for the oxidation of benzaldehyde into benzoic acid in non-β-oxidative pathway. In *P. mume*, 16 *PmBALD* homologs were achieved with the snapdragon *BALD* as the query sequence. Contrasted with snapdragon, *PmBALDs* showed low correlation with the emission of benzenoids with the max TPM of 2,296. Finally, 32 candidate genes were angled homologous to *CbBEAT* hosting the direct step toward benzyl acetate, and 5 *PmBEATs* genes were found positively expressed with this component.

### Identifying DEGs for cytochrome P450 and SDR families

Many genes of the large enzyme families appeared to flaunt their expressions in the transcriptome profiles using MapMan.

P450 proteins were detected notably arising in the blooming stage within a total of 201 cytochrome P450 genes in *P. mume*. Moreover, two P450 genes were highlighted in the up-regulated DEGs with a fold change of more than 18. One gene, predicted as cytochrome P450 82D47, was homologous to Cytochrome P450 82A4 in *Cajanus cajan*, of which the TPM ranged from 2,381 to 45,146; the other was isoleucine N-monooxygenase 2 (cytochrome P450 79A68), with a TPM ranging from 605 to 20,604.

The short-chain dehydrogenases/reductases (*SDR*) family attended many reactions to convert the aldehyde groups, which might participate in the formation of benzyl alcohol. In the genome of *P. mume*, 147 *SDR* related genes with various expression levels were retrieved, and 9 candidate genes were gained, which were highly expressed in the flower. Among these candidate *SDR*s, three genes increased to the level of 10,000 TPM in the blooming stage, two around a TPM of 10,000, but in other four organs, the TPM of these genes approached zero or remained low. Though one gene was significantly identified in the blooming stage, higher than the former four genes, there still remained around 4,000 TPM in fruits, leaves, roots or stems. The other four genes peaked in the flower with TPMs under 3,300.

### Transcription factors out of DEGs

**Figure 8:**
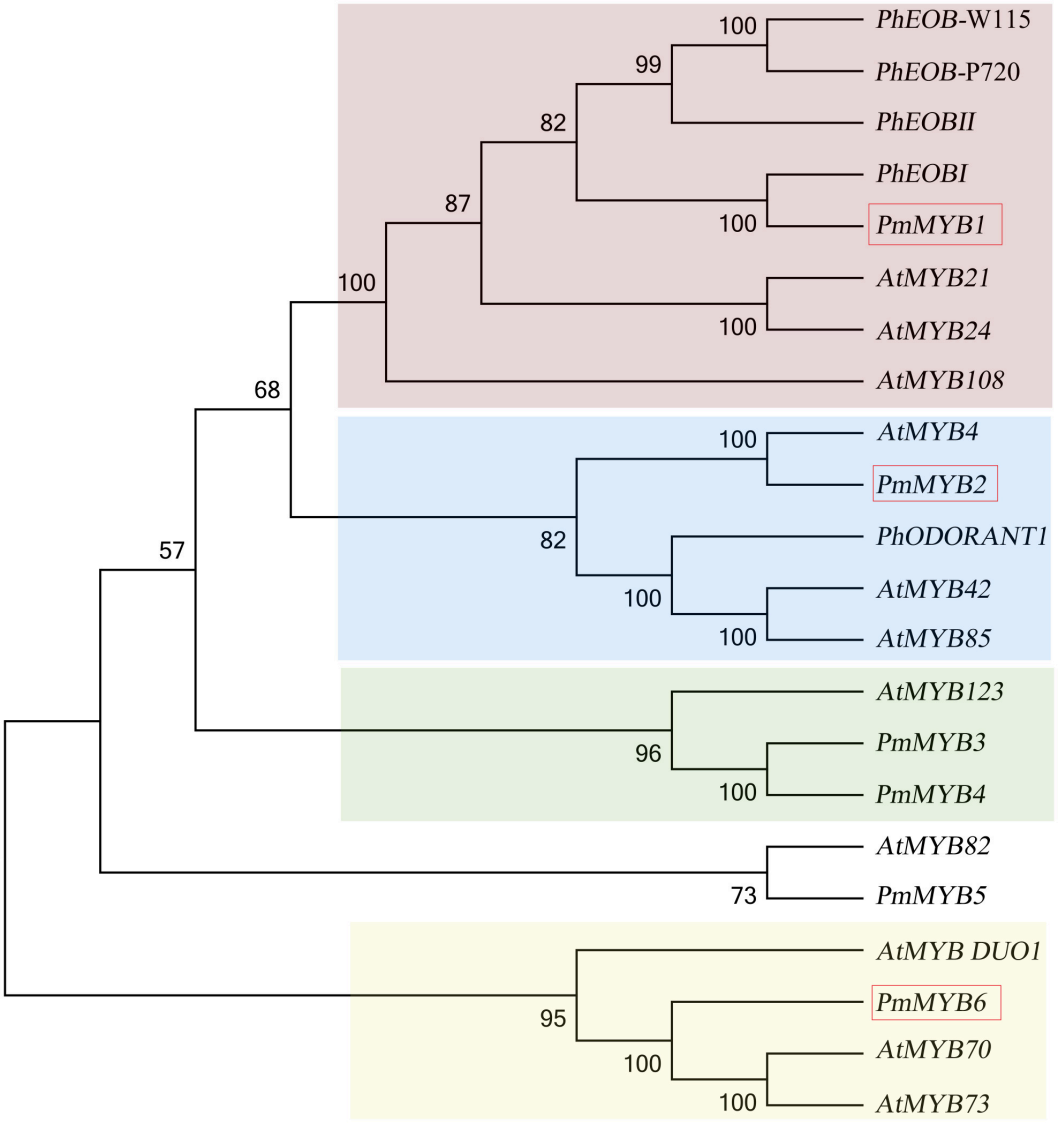
**Phylogenetic analysis of MYB transcription factors**. TFs in red box mean high expression in blooming stage.

Transcription factors are believed to be essential in controlling many secondary metabolic processes. In our comparative transcriptome studies for the scent formation of Mei flower, we identified 595 TFs (transcriptional regulators included) of 76 TF families classified by PlantTFDB database, representing 7.61% of all DEGs (Supplementary Table 6). Most TF gathered in MYB family (50, 8.4%), followed by bHLH (42, 7.05%), and NAC (35, 5.88%). Among the flower specifically expressed genes (profile 46), there were 36 TFs (6% of 595) distributed in 18 TF families, containing MYB-related (6), MYB (6), NAC (3), etc. MYB family was first discovered in the synthesis of flower scent (Figure 8). Phylogenetic analysis illustrated there was one *PmMYB1*, homologous to the flower-specific *PhEOB1* reported in petunia. *PhEOB*s and *PmMYB1* fell into the same branch together with *AtMYB21* and *AtMYB24*. And in the next branch, *PmMYB2* homologous to *AtMYB4* was clustered with *PhODORANT1. PmMYB3*, and *PmMYB4* were homologous to the *AtMYB123* (*TT2*). As for the expression, three genes (*PmMYB1*, PmMYB6, and PmMYB2) assumed high TPMs in the blooming stage.

### Gene expression validation

**Figure 9:**
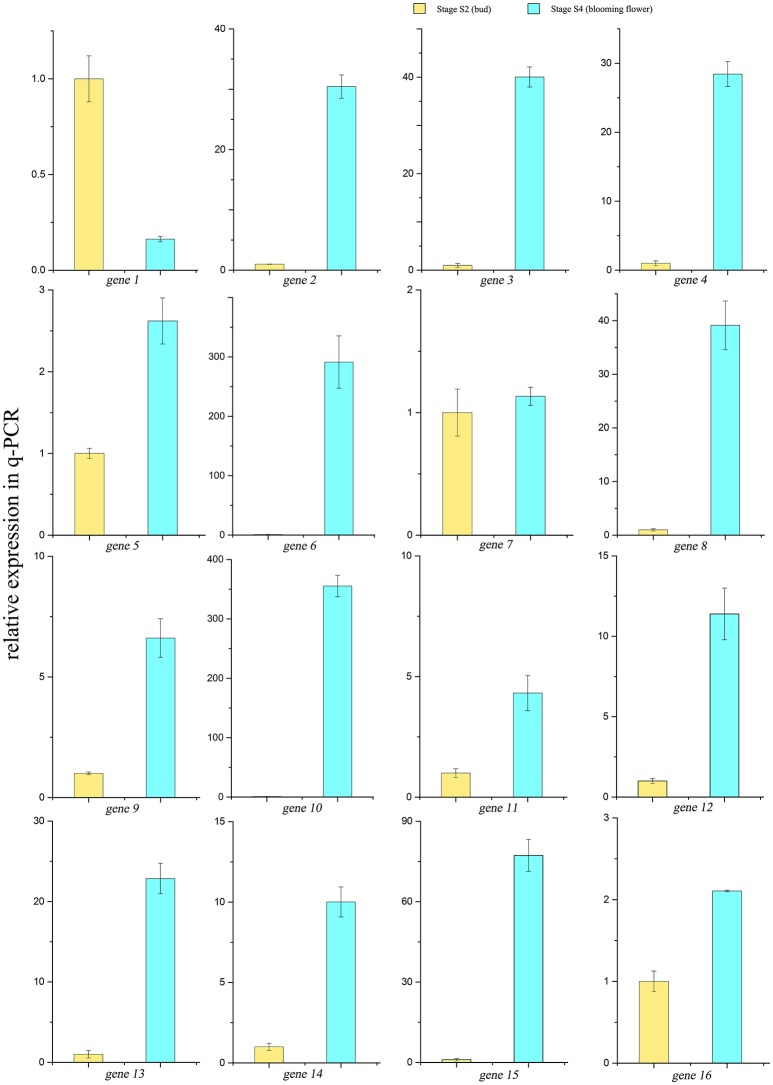
**qRT-PCR results of 16 genes in the transcriptome**. Yellow bars showed the relative expressions of S2 stage, blue ones showed expressions in the S4 stage.

**Figure 10:**
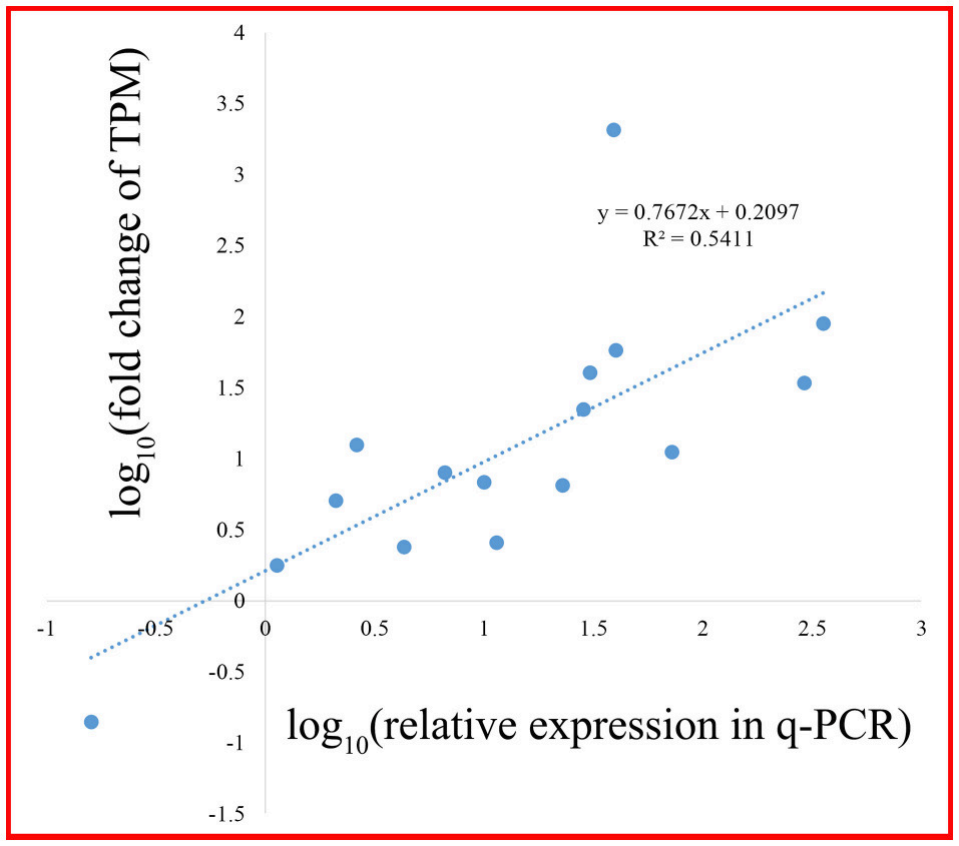
**Correlation analysis of fold change between q-PCR and RNA-seq**.

A scale of genes related shikimate and benzenoid biosynthesis was selected to test the expression profiles during flower development using real-time quantitative PCR (qRT-PCR) analysis (part results not shown). Sixteen genes in the transcriptome were chosen randomly (Figure 9). The genes‘ expression profiles were primarily coincident with those shown by RNA-seq in tendency and multiplication, though the expression in these genes existed huge difference. The linear regression analysis confirmed the results between q-PCR and RNA-seq and revealed a positive correlation with R^2^ of 0.54 (Figure 10). These results demonstrated the credibility of sequencing data generated and the pattern profiled in this study.

## Discussion

Mei trees, since their origin in China, have been extensively cultivated in Asia. and an assembled 280 Mb reference genome accelerated the further researches for plant characterization (Zhang et al., 2012). The previous results indicated that benzenoids were the dominant volatile compounds serving as the core advantage for *Prunus* species (Hao et al., 2014a). Even though benzenoids are one of the most interesting natural product, the molecular mechanism behind their synthesis is still unknown. Based on 7,813 DEGs achieved from comparative transcriptome analysis, 681 scent-related DEGs specially expressed in flowers were sifted. The mechanism of floral scent formation was gradually explored after the separation of core TFs, specifically the MYB family TFs. These shifty candidate genes offered a bird eye view for the formation of floral fragrance in *P. mume*, thereby constructing a latent network to regulate the structural genes of floral scent. With the scent controlling genes obtained, this transcriptome would lay the basis for revealing biosynthesis pathway of benzenoids, particularly benzyl alcohol and benzaldehyde which acted as the vital precursor substance of principal aroma in Mei flower and was enriched in the species of genus *Prunus*.

### Benzaldehyde set a core anchor in benzenoids of *P. mume*

Benzaldehyde is very familiar compound, however, its biosynthesis remains an unresolved issue. Numerous attempts have been made to unmask the truth for a long time, which maybe is the cause of diversification in benzenoids (Croteau, 1978). As for as the scent composition of Mei flower is concerned, benzyl acetate accounted for 90.4% of the floral volatile compounds, but benzaldehyde possessed 83% share in the endogenous compositions (Hao et al., 2014b). Indeed, the content of benzaldehyde was far larger, almost 7-fold, than that of benzyl acetate. As reported in the petunia and other species, benzaldehyde comes from the phenylalanine by means of isotope labeling (Boatright et al., 2004). Andcinnamoyl-CoA hydratase/ lyase is suspected to catalyze hydration and cleavage of cinnamoyl-CoA to benzaldehyde and acetyl CoA in *Hypericum androsaemum* (Abd El-Mawla and Beerhues, 2002). CoA ligases seem not to been involved in the production of benzaldehyde in petunia. The suppression of *Ph4CL* does not affect the benzenoid spectrum, and there are no changes in benzaldehyde but the components related to BA undergo changes after the RNA interference suppression of *PhCNL* (Klempien et al., 2012). However, the results of transcriptome in *P. mume* displayed that the expressions of *PmPALs* and *PmCNL* were significantly detected in the benzaldehyde production stage, suggesting that the formation of endogenous benzaldehyde might have converged from two paths in the flower of *P. mume*.

### Cytochrome P450 family expressed spatially to form benzaldehyde

With up to 1% of total gene annotations of each plant species, plant cytochrome P450s catalyze plentiful monooxygenation/ hydroxylation reactions in primary and secondary metabolism (Mizutani and Ohta, 2010). In *P. mume*, two P450s are reported to coexpress to produce the cyanogenic glycosides prunasin and amygdalin (MAN), which originated from phenylalanine (Yamaguchi et al., 2014). CYP79D16 is responsible for the conversion of phenylalanine into phenylacetaldoxime (PAOx) particularly in seedlings. CYP79A68 existed in the buds with the similar structure and was up-regulated in the comparative transcriptome. This drove us to suspect that those two may be controlled to get different functions in different organs. CYP71AN24 has broad substrate specificity, mainly converting PAOx into MAN. After the degradation of MAN, benzaldehyde is released. As a whole in benzenoid pathway, P450s might cleave a new path to the benzaldehyde.

### SDR family may count for the benzyl alcohol

The SDR genes participate in many aspects of primary and secondary metabolism and are noteworthy in carbonyl-alcohol oxido-reduction (Tonfack et al., 2011). These genes possess at least two domains binding NAD and substrates, respectively, and the latter domain determines the specificity of substrate (Moummou et al., 2012). During the transformation of the phenylalanine, SDR family plays a vital role in the benzenoid volatiles. In the flowers of Damask roses, 2-Phenylethanol (2PE) is biosynthesized from phenylalanine via intermediate phenylacetaldehyde under the catalysis of *PAR*, which is classified as short-chain dehydrogenase reductase (Chen et al., 2011). The presence of highly expressed *PmSDRs* homologous to PAR satisfied the requirement of benzyl alcohol. And the benzaldehyde was predicted to be transformed via the same oxido-reductive method as the formation of 2PE. The deep research of SDR family may contribute to unravel the accumulation of benzaldehyde in the *Prunus*.

### Heterologous regulation of the benzenoid biosynthesis in woody plant

To date, only a few TFs have been identified in plants to answer the formation of floral scent. There are four MYB members in petunia to regulate benzenoid volatiles (Muhlemann et al., 2014). *PhODO1* was the first transcription factor found to promote benzenoid biosynthesis at the stage of scent formation in petunia (Verdonk et al., 2005). This TF functions in the shikimate pathway toward benzenoids by accelerating the core structure enzymes like DHAPS, EPSPS, and PAL. Then, two *PhEOBs* are found to control the downstream benzenoids, not affecting the color formation (Spitzer-Rimon et al., 2010). Together with these three TFs, a complex feedback loop has been demonstrated to contain positive and negative effects. In *P. mume*, several gateway genes aroused exceedingly, such as *PmDAHPSs, PmADTs*, and *PmPALs*, implying the similar regulation method in scent production with petunia. However, there is one ODO-like genes and one EOB-like gene up-regulated in the comparative transcriptome and the kind of structure genes are not all the same as what appears in petunia. These features might distinguish *P. mume* from petunia for scent production no matter in quantity of volatiles or in aroma types. Despite the high expression structure genes and flower specificity, there appears considerable difference in scent regulation for this woody plant.

## Conclusions

In comparative transcriptome, vital differences of gene expression between bud stage and squaring stage were observed, DEGs with transcriptomes of different organs were found, which provided a special view of floral scent formation in *P. mume*. Moreover, RNA-seq was helpful to discover the latent scent-related genes in this dicot. Among them, flower-specificP450 genes and *PmSDRs* might function in the prime steps controlling the flux into some key precusors like benzaldehyde and benzyl alcohol. All TFs in the DEGs were discerned and classified. Therefore, *PmMYBs* might master the biosynthesis of the floral volatile benzenoids together some unknown TFs.

All these results furnish practical information to explore the induction for floral scent formation and regulation in *P. mume*. Meanwhile, it also offers us the chance in directional manipulation of volatile spectrum in *Prunus* to enhance human friendly living condition or commercial values for fruit production. Perhaps the more interesting questions arising from this research are the identification of exact genes contributing to the specification of benzaldehyde and benzyl alcohol, and how those genes are regulated by the TFs. In-depth studies are required to answer these questions and to provide an insight into the regulatory network and induction mechanism of scent woody plants.

## Author contributions

KZ and WY contributed equally to this work. KZ, WY, and QZ designed the whole experiments. KZ wrote the manuscript. KZ, YZ, YL, JZ, and SA analyzed the data. All authors read and approved the final manuscript.

### Conflict of interest statement

The authors declare that the research was conducted in the absence of any commercial or financial relationships that could be construed as a potential conflict of interest.

## Supplementary material

Supplementary Figure 1 **Quality score from Fast QC**.

Supplementary Figure 2 **Scatter plot of different expression genes**.

Supplementary Figure 3 **DEGs annotation by mercator**.

Supplementary Table 1 **Primer sequences for qRT-PCR**.

Supplementary Table 2 **The candidate genes in the benzenoid pathway**.

Supplementary Table 3 **The up-regulated genes of DEGs in Mei flower**.

Supplementary Table 4 **The down-regulated genes of DEGs in Mei flower**.

Supplementary Table 5 **GO enrichment analysis of DEGs**.

Supplementary Table 6 **TF list in the DEGs**.
