# Supplementary figures and images for: Comparative Transcriptome Reveals Benzenoid Biosynthesis Regulation as Inducer of Floral Scent in the Woody Plant Prunus mume
Source: Front Plant Sci. 2017 Mar 10;8:319. doi: 10.3389/fpls.2017.00319 (PMC5345196; doi:10.3389/fpls.2017.00319)

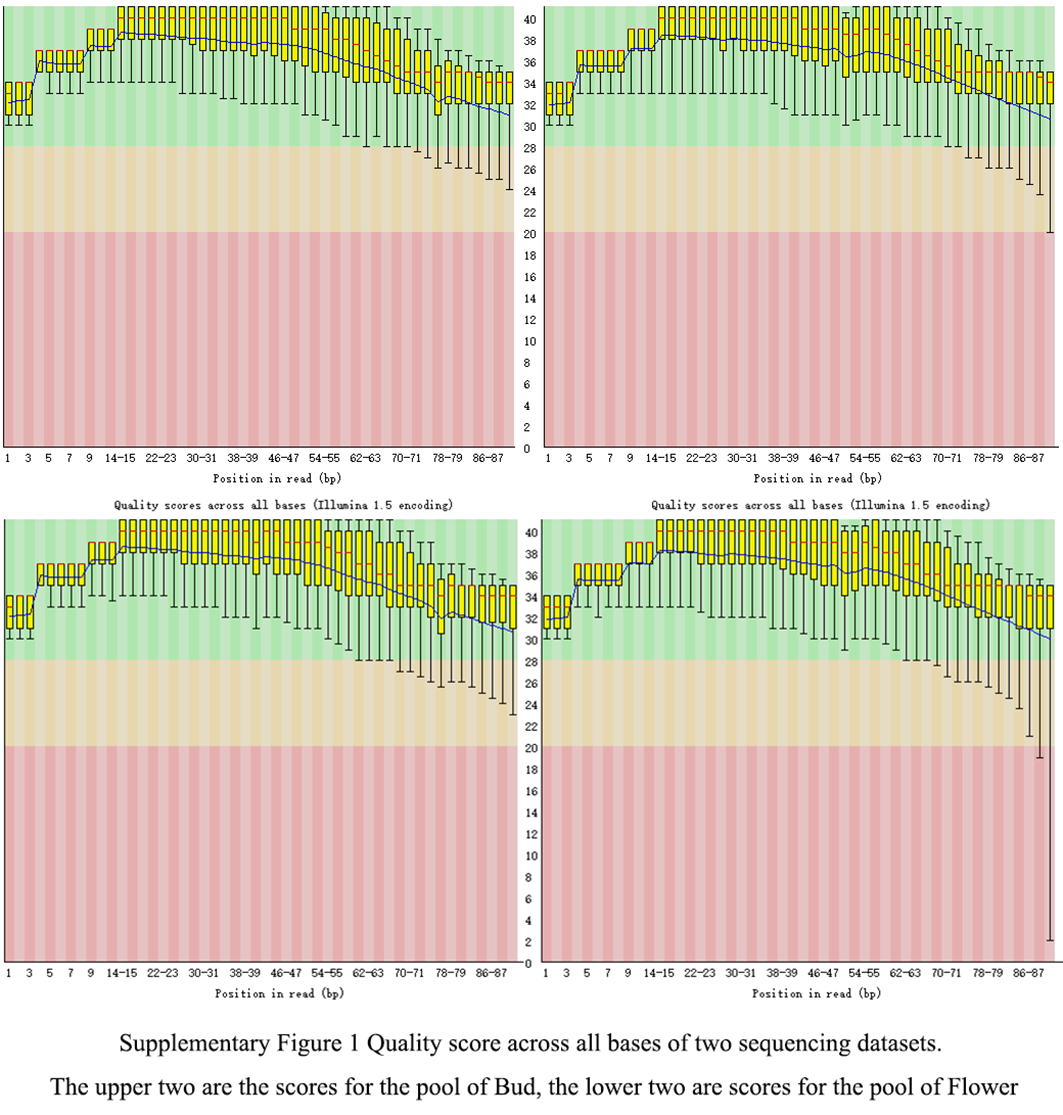

Supplement: Supplementary Figure 1 — Quality score from Fast QC. [file Image1.TIF]

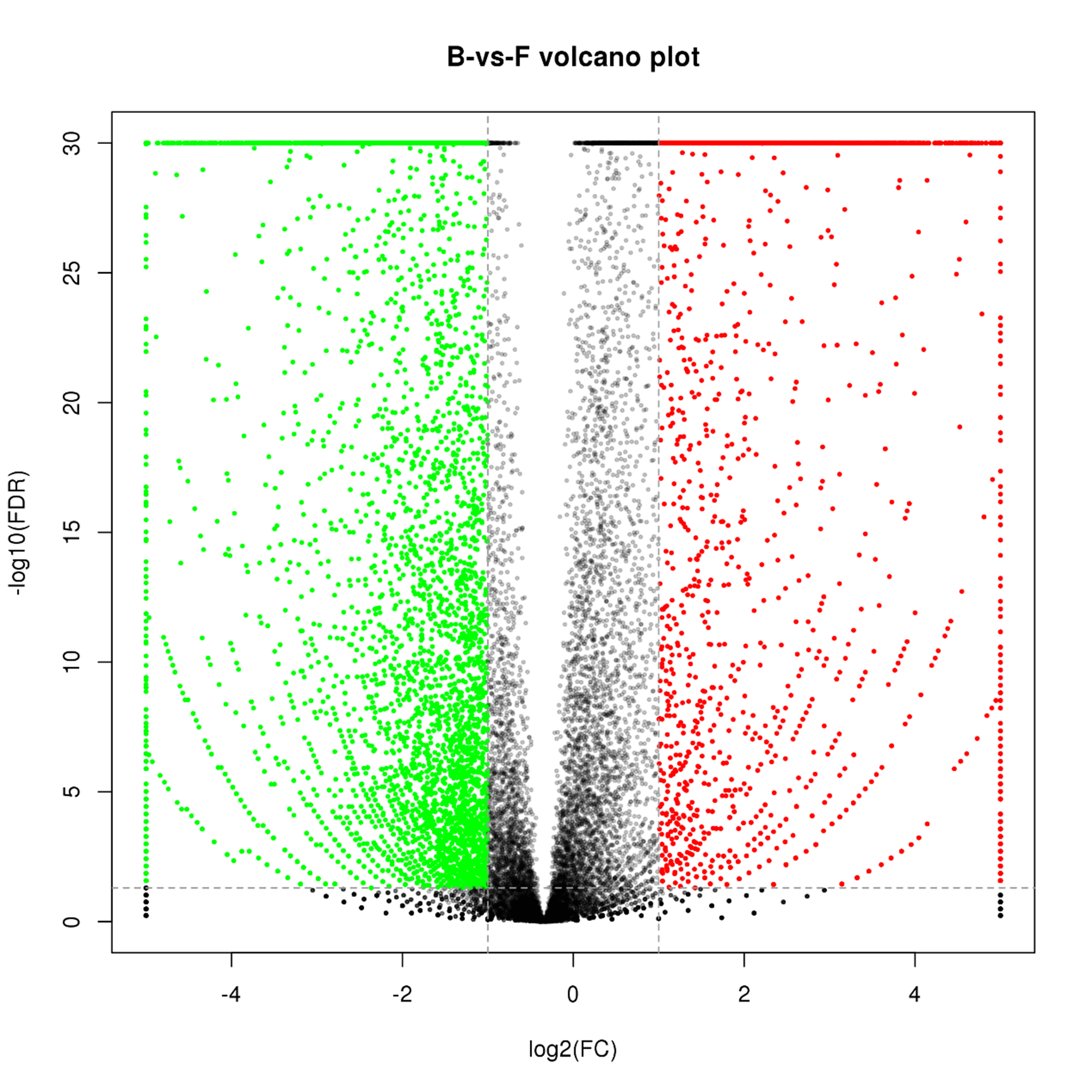

Supplement: Supplementary Figure 2 — Scatter plot of different expression genes. [file Image2.TIF]

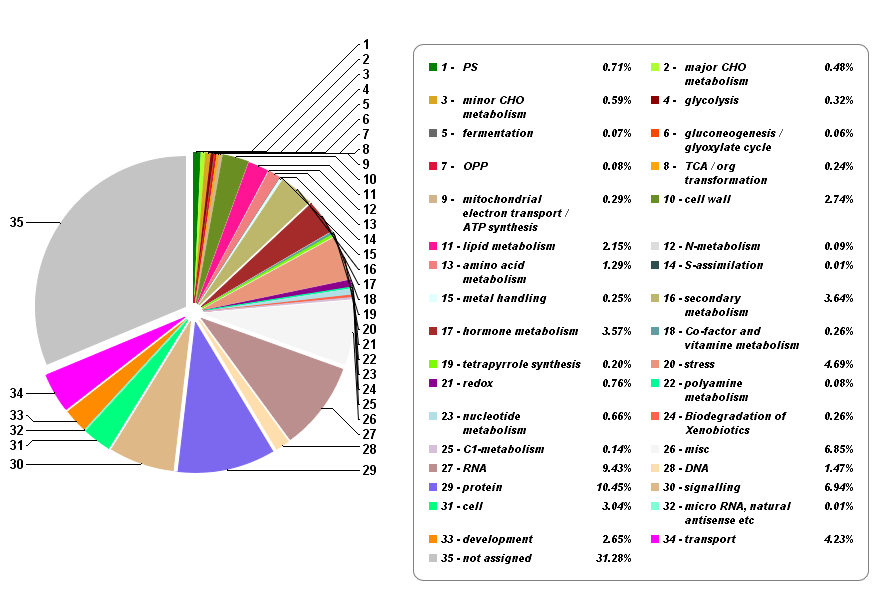

Supplement: Supplementary Figure 3 — DEGs annotation by mercator. [file Image3.PNG]
